# 

**Published:** 1843-09-16

**Authors:** 


### CONTENTS.

A Course of Clinical Lectures, delivered at the Hôtel Dieu, Paris, for the Session 1842–'43. By A. F. Chomel, M.D. Lecture 12, - - - - - - 205

Case of Hemiplegia consequent on tying the common carotid. By O. Fairfax, M.D. - - - - - - - - 208

On Sulphuretted Hydrogen Gas as the cause of Malarious Fevers, - - 208

*Bibliographical Notices.*

Address delivered before the American Society of Dental Surgeons, at the opening of its Fourth Annual Meeting. Baltimore, July 18th, 1843. By Chapin A. Harris, A.M. M.D. &c. - 209

Physiology Vindicated, in a Critique on Liebig's Animal Chemistry. By Chas. Caldwell, M.D., - - - - - 210

*Retrospect of the Medical Sciences.*

Morbid condition of the Blood in Glanders, - - - - - - - 210

Successful case of Transfusion of Blood. By Dr. Prichard, - - - - - 211

Statistics of Epilepsy. By M. Leuret, 211

The Diagnosis of Uterine Diseases, 211

New Test for Arsenic, - - - 212

Emplastrum Cerati Saponis, - - 213

Case in which a foreign body was lodged in the right bronchus, - - - 213

Influence of Rickets upon the growth of the Skull. By A. Shaw, M.D. - 214

Medicinal use of Saffron, - - - 214

Dr. Percy on Diabetes Mellitus, - - 215

Agencies influencing the circulation and condition of the blood in Reptiles, 215

Diagnosis of Paraplegia, - - - 215

New Method for obtaining Milk, - 215

Voyage up the Mediterranean in the treatment of Phthisis, - - - - - 215

Oxalic Acid, - - - - - - 216

Cause of Bruit de Soufflet in Anæmia, 216

Physical condition of the blood in plethora, 216

Prognosis in cases of Uterine Hemorrhage, 216

Stomatitis Ulcerosa, - - - 216

